# Electrochemistry of the Self-Assembled Monolayers of Dyads Consisting of Tripod-Shaped Trithiol and Bithiophene on Gold

**DOI:** 10.3390/molecules190915298

**Published:** 2014-09-24

**Authors:** Toshikazu Kitagawa, Hiroaki Matsubara, Takao Okazaki, Koichi Komatsu

**Affiliations:** 1 Department of Chemistry for Materials, Graduate School of Engineering, Mie University, Tsu, Mie 514-8507, Japan; 2 Institute for Chemical Research, Kyoto University, Uji, Kyoto 611-0011, Japan

**Keywords:** self-assembled monolayer, molecular tripod, bithiophene, adamantane, cyclic voltammetry

## Abstract

Self-assembled monolayers (SAMs) of tripod-shaped trithiols, consisting of an adamantane core with three CH_2_SH legs and a bithiophene group, were prepared on a Au(111) surface. Adsorption in a tripod-like fashion was supported by polarization modulation-infrared reflection absorption spectroscopy (PM-IRRAS) of the SAMs, which indicated the absence of free SH groups. Cyclic voltammetry showed an irreversible cathodic wave due to reductive desorption. The SAM also showed an anodic wave due to the single-electron oxidation of the bithiophene moiety without concomitant desorption of the molecules. Although oxidation was irreversible in the absence of a protecting group, it became reversible with the introduction of a terminal phenyl group. The charge of the oxidation was one-third that of the reductive desorption, confirming a three-point adsorption. The surface coverage was *ca*. 50% of that expected for the *anti* bithiophene conformation, which suggested that an increase in the surface area per molecule had been caused by the presence of an energetically high-lying *syn* conformer. In accordance with this, the line shape of the oxidation wave suggested an electrostatic repulsive interaction between neighboring molecules.

## 1. Introduction

The tight binding of a sulfur atom in an organic molecule to a metal gold surface provides a secure and facile way to prepare a self-assembled monolayer (SAM) [1,2,3,4], which allows a reliable means for interface functionalization. A variety of monolayers, prepared from properly designed thiols and bisulfides, have been extensively studied for applications to sensors [5,6,7], molecular machines [8,9,10,11] and molecular electronic devices [12,13,14,15,16,17,18,19,20,21,22].

**Figure 1:**
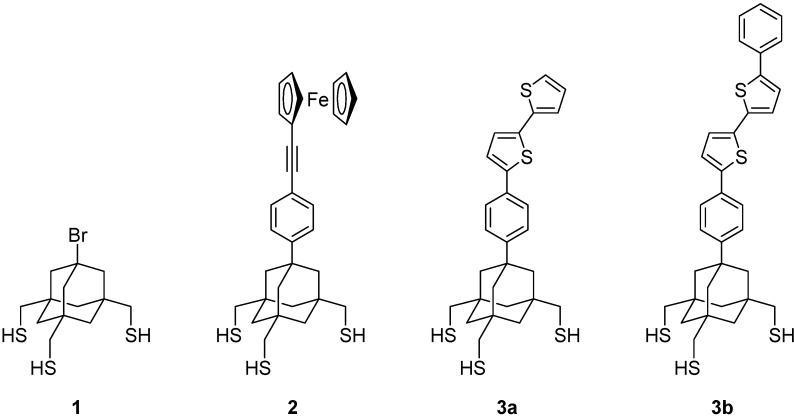
Structures of tripod-shaped trithiols with an adamantane core. The structures of **3a** and **3b** represent *anti* conformers.

Tripod-shaped molecules that have three legs with a terminal sulfur group at the end of each leg can serve as tight-binding anchors for the construction of SAMs [8,17,18,19,20,21,22,23,24,25,26,27,28,29,30,31,32,33,34]. Previously, we reported that molecular tripod **1**, composed of a rigid adamantane core and three CH_2_SH legs [23,24,27], forms a SAM on Au(111) with a highly ordered arrangement by three-point adsorption (Figure 1). We further succeeded in preparing a trithiol with terminal ferrocene **2**, in which the ferrocenyl group was connected with an adamantane tripod through a linear phenyleneethynylene linker [25,26,27]. Due to the area-demanding tripod structure, **2** enabled the formation of a SAM with “ideal” behavior, where no electrostatic interaction among surface ferrocenyl groups was present, which is otherwise difficult to realize without the help of non-electroactive diluting molecules.

**Figure 2:**
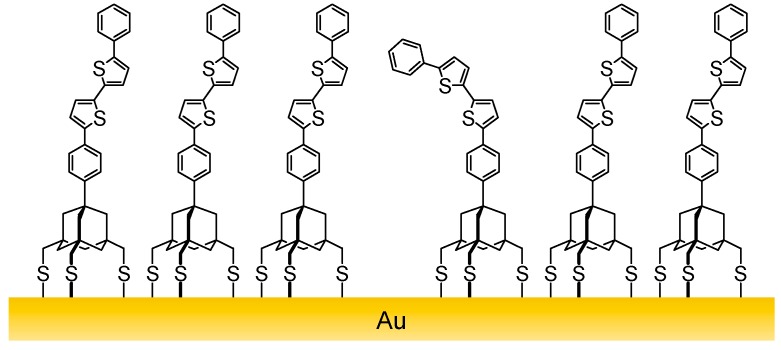
Schematic representation of a SAM of *anti*-**3b** containing the *syn*-conformer.

In the present study, we synthesized dyads **3a** and **3b**, which consist of another electroactive molecule, bithiophene, connected with the adamantane tripod, and investigated the electrochemistry of their SAMs. SAMs of oligothiophenes with a monodentate anchor [20,35,36], bidentate anchor [37] or triarylmethyl tripod anchor [17,18,19,20,21,22] have been used in the construction of molecular electronic devices, such as photovoltaic cells and light-emitting diodes. Oligothiophenes with an all-*anti* (all-transoid) conformation can adopt a near-perpendicular orientation in SAMs, while the presence of a *syn*-linkage could prevent dense packing because of its bent structure. Since **3a** and **3b** have no alkyl side chains on the thiophene rings, their *anti* conformers form SAMs with potentially the same surface density as that of **1** and **2**. Thus, reduced surface coverages of tripods **3**, if observed, could be good indicators for the presence of *syn*-linked bithiophene in SAMs (Figure 2).

## 2. Results and Discussion

### 2.1. Synthesis of Tripodal Trithiol–Bithiophene Dyads

**Scheme 1:**
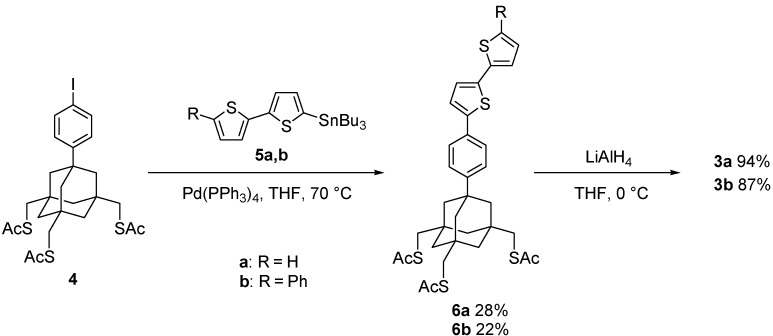
Synthesis of trithiols **3a** and **3b**.

Tripod-linked bithiophenes **3a** and **3b** were prepared via the Stille coupling of *p*-iodophenyl tris(thioacetate) **4** [23,26] with the corresponding 2-tributylstannylated bithiophenes, **5a** and **5b**, followed by deprotection of the thiol groups (Scheme 1). Unlike the good-to-fair yields that have been reported for the Stille coupling of stannylbithiophene [38,39,40], the reactions with **5** were relatively poor, which could be ascribed to possible catalyst poisoning by the sulfur atoms in the legs. Reduction of the acetylthio groups of **6** by LiAlH_4_ afforded the target trithiols **3a** and **3b** in good yields. The reactions were so clean, that the products could be obtained in a pure form with no purification (**3a**) or after simple flash chromatography (**3b**). Further purification by HPLC was not beneficial, owing to a significant loss of the sample, due to the immobilization of the molecules on the inner surface of the stainless-steel column during separation.

### 2.2. Formation of the SAM of **3** and Characterization by PM-IRRAS

The SAMs of tripods **3a** and **3b** were prepared by immersing a Au(111) substrate, prepared by depositing gold on a mica sheet, into 0.1 mM solutions in ethyl acetate (**3a**) or dichloromethane (**3b**) at ambient temperature for 24 h or longer. The SAM-modified substrate was washed thoroughly with the same solvent and air-dried.

**Figure 3:**
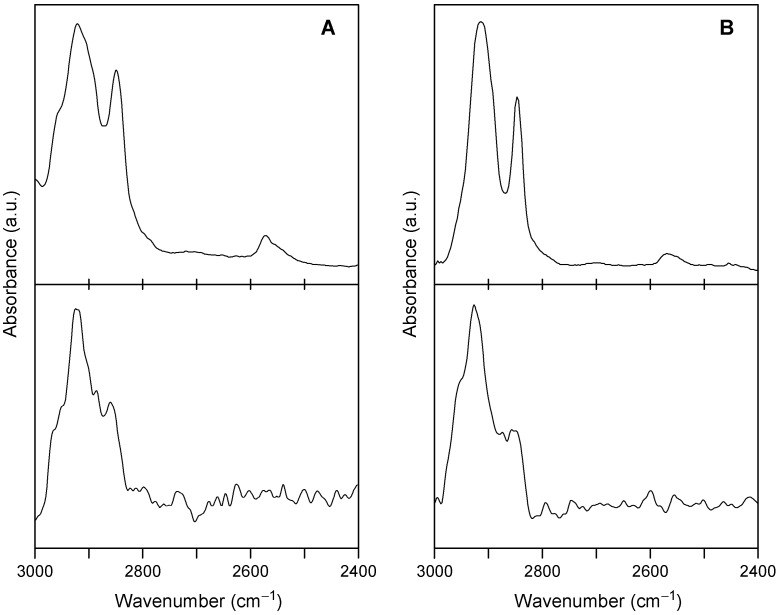
Infrared absorption spectra of trithiols **3a** (**A**) and **3b** (**B**): upper spectra, transmission spectra for drop-cast films prepared on a KBr plate using CHCl_3_ solutions; lower spectra, polarization modulation-infrared reflection absorption spectroscopy (PM-IRRAS) spectra for the thiolate SAMs on Au(111).

The infrared reflection absorption spectrum (IRRAS) of the SAMs, recorded in a polarization modulation mode (PM), showed no distinct peak of the S–H stretching vibration, which was seen at *ca*. 2570 cm^−1^ in the infrared spectrum of the neat samples of **3** (Figure 3). This is consistent with the formation of SAMs using all three sulfur legs, which leads to a perpendicular orientation of the molecules. The peaks of antisymmetric and symmetric CH_2_ stretching, which were observed at around 2915 and 2848 cm^−1^, respectively, for neat samples, were shifted to higher wavenumbers by 7–11 cm^−1^ in the SAMs. These shifts might indicate the tightening of the C–H bonds due to the fixation of the three legs on the Au surface.

### 2.3. Electrochemistry of the SAM of **3**

#### 2.3.1. Reductive Desorption

**Figure 4:**
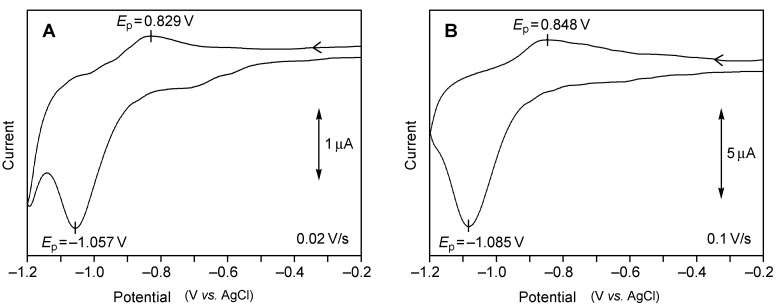
Reductive desorption of the SAMs of **3a** (**A**) and **3b** (**B**) on Au(111), as observed by cyclic voltammetry using the surface-modified gold substrate as a working electrode in 0.5 M aqueous KOH: geometric area of the working electrode, 0.152 cm^2^; scan rate, 0.02 V·s^−1^ for **3a** and 0.1 V·s^−1^ for **3b**.

The cyclic voltammetry of the SAMs of **3a** and **3b** in aqueous KOH (Figure 4) showed irreversible reduction peaks at −1.057 and −1.085 V (*vs.* Ag/AgCl), respectively, due to the reductive desorption of the thiolate ion [41,42,43,44,45,46,47]:

RS–Au(s) + e^−^ → RS^−^ + Au(s) (1)

A small oxidation wave at approximately −0.84 V was also observed due to a readsorption of the thiolate ion [43,44,45].

The peak potential (*E*_p_), the charge of the reductive wave (*Q*_red_) and the full width at half-maximum (Δ*E*_fwhm_) are listed in Table 1, together with the values for related SAMs. The SAMs of long-chain *n*-alkanethiols are known to show a large negative *E*_p_ (<−1.0 V), because the adsorbed molecules resist desorption due to strong attractive interactions between van der Waals-contacting alkyl groups [46]. On the other hand, the large negative *E*_p_s for the SAMs of tripod molecules 1–3 should be rather attributed to the tight binding of the molecules to the substrate by the three S–Au bonds.

**Table 1:** Peak potential (*E*_p_), charge (*Q*_red_) and full width at half-maximum (Δ*E*_fwhm_) for the reductive desorption of thiol SAMs on Au(111), as measured by cyclic voltammetry *^a^*.

Thiol	*E*_p_	*Q*_red_ *^b^*	Δ*E*_fwhm_
	(V *vs.* Ag/AgCl)	(µC·cm^−2^)	(mV)
**1** *^c^*	−1.088	100	91
**2** *^d^*	−0.977	91	125
**3a**	−1.057	51	108
**3b**	−1.085	49	121
*n*-C_12_H_25_SH *^c^*	−1.084	100	20

*^a^* In 0.5 M aqueous KOH; *^b^* calculated from the reduction peak area. The uncertainty due to sample-to-sample variations is ±10%; *^c^* [23]; *^d^* [26].

The small Δ*E*_fwhm_ for *n*-dodecanethiol can also be ascribed to the strong attractive interaction between neighboring alkyl chains [46], while the much larger values observed for tripod trithiols indicate the insignificance of such interactions.

The surface coverage of the adsorbed molecules (Γ_red_) is proportional to *Q*_red_:

Γ_red_ = *Q*_red_/*nFA* (2)

Here, *n* = 3 electrons per molecule, *F* is the Faraday constant and *A* is the geometric area of the electrode (0.152 cm^2^). As indicated in Table 1, we previously reported [23,26] that the SAMs of bromine- and ferrocene-terminated trithiols (**1** and **2**) showed *Q*_red_s of *ca*. 100 µC·cm^−2^, which approximated that observed for *n*-dodecanethiol. On the other hand, the reductive charges of **3a** and **3b** were approximately 50 µC·cm^−2^, which may be explained by the greater area per molecule due to the bulky bithiophene moiety.

**Figure 5:**
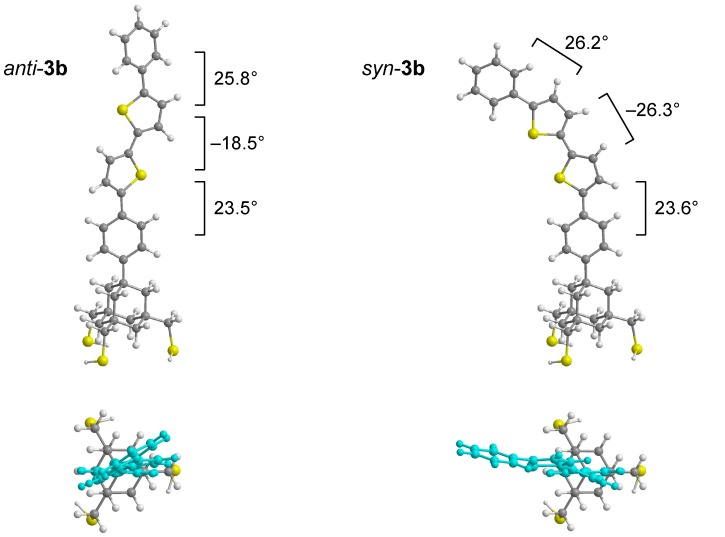
DFT-optimized structures of *anti* and *syn* conformers of trithiol **3b** calculated at the B3LYP/3-21G(d) level. The dihedral angles between adjacent rings are shown. Lower structures represent top views, with the diphenylbithiophene moiety highlighted in light blue.

Figure 5 shows the density functional theory (DFT)-optimized structures of **3b** with *anti* and *syn* conformations. The former had a near-perpendicular structure, while the latter was bent to a large extent and was 1.5 kJ·mol^−1^ higher in Gibbs energy than the former [48]. The dihedral angles between the mean planes of adjacent benzene and thiophene rings were constant within a range of 23°–27° in both structures, while those between two directly bound thiophene rings showed a large difference (*anti*: 18.5°, *syn*: 26.3°), due to the steric repulsion between the hydrogen atoms in *syn*-bithiophene. The *anti* conformation would be more favorable in SAMs, because of not only its lower energy, but also its small occupied area, which allows for higher surface coverage. However, the small *anti*-*syn* energy difference may allow some fraction of molecules to adopt the *syn* conformation.

Since adsorbate **3a** is less bulky than **3b**, the observed similar coverages of their SAMs would be due to either a greater *syn*/*anti* ratio or less complete adsorption of **3a**. However, the latter possibility can be ruled out, because the surface coverage was not changed by prolongation of the SAM preparation time.

#### 2.3.2. Oxidation of the Bithiophene Moiety

**Figure 6:**
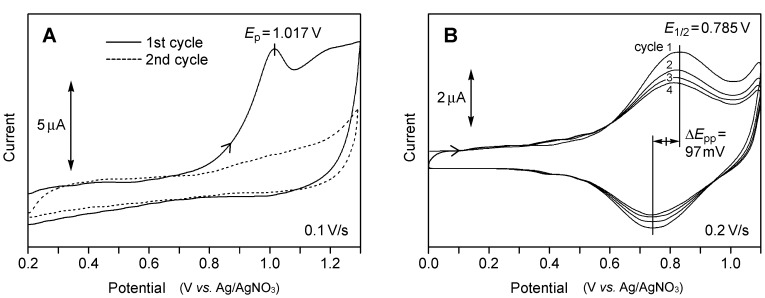
Cyclic voltammograms of the SAMs of **3a** (**A**) and **3b** (**B**) recorded in CH_2_Cl_2_: geometric area of the working electrode, 0.152 cm^2^; scan rate, 0.1 V·s^−1^ for **3a** and 0.2 V·s^−1^ for **3b**. Tetrabutylammonium perchlorate (TBAP) (0.1 M) was used as a supporting electrolyte.

Cyclic voltammetry was performed in a positive potential range in CH_2_Cl_2_ to examine the oxidation process of the bithiophene moiety. The SAM of **3a** showed an irreversible oxidation wave at 1.017 V (*vs.* Ag/AgNO_3_), corresponding to single-electron oxidation of the bithiophene moiety (Figure 6A). This wave disappeared in the second cycle, but a subsequent voltammetry over −0.2 to −1.2 V in aqueous KOH using the same substrate showed a reductive wave (*Q*_red_ = 48 µC·cm^−2^) similar to that in Figure 4B (data not shown). These results rule out desorption during electrochemical oxidation and suggest that the bithiophene group underwent decomposition after single electron oxidation. Since oxidative coupling is prohibited for a surface-confined monolayer, it is more likely that the radical cation was captured by molecular oxygen, followed by ring cleavage [49]. The peak area of the oxidative wave corresponded to a charge of 16 ± 2 µC·cm^−2^.

An oxidative scan of the SAM of **3b**, however, showed a reversible wave at *E*_1/2_ = 0.785 V (*vs.* Ag/AgNO_3_) with a charge of 17 ± 2 µC·cm^−2^ (Figure 6B). The anodic to cathodic peak separation (*E*_pp_ = 97 mV) and the full width at half-maximum (*E*_fwhm_ = 260 ± 20 mV) were considerably greater than those expected for an “ideal” Nernstian redox system: *E*_pp_ = 0 and *E*_fwhm_ = 3.53 *RT*/*nF* (90.6 mV at 25 °C and *n* = 1) [50]. This was in contrast to the result for the SAM of 2, which demonstrated ideal behavior as a result of the absence of the electrostatic interaction between neighboring ferrocenyl groups [26]. Large *E*_pp_s and *E*_fwhm_s have been commonly observed for other densely packed electroactive SAMs due to lateral molecular interaction [51,52,53]. In the present SAMs, such an interaction would be enhanced by the presence of the area-demanding *syn* conformer.

In Figure 6B, a *ca*. 7% decrease in the peak current was observed with each cycle. This is probably due to the partial desorption of the molecule, since a reductive desorption measurement after the fourth cycle showed a charge that was 40% less than that observed in Figure 4A [54].

**Figure 7:**
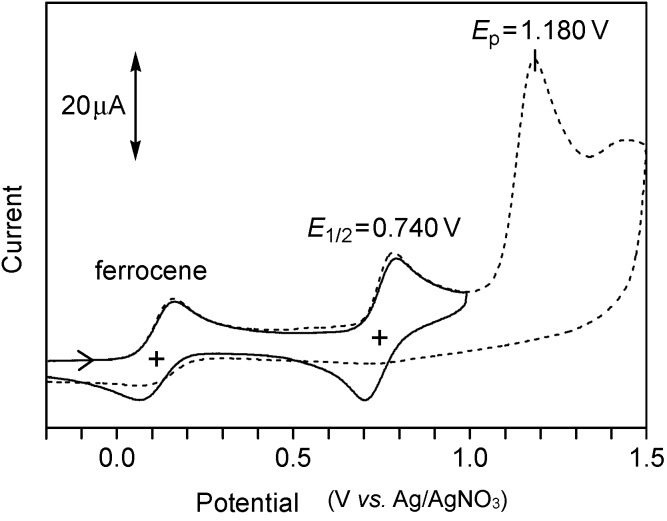
Cyclic voltammograms of a CH_3_CN solution of **6b** (1 mM) recorded over two different potential ranges. Ferrocene (1 mM) was added as a current standard: scan rate, 0.1 V·s^−1^; working electrode, glassy carbon; counter electrode, Pt wire. TBAP (0.1 M) was used as a supporting electrolyte.

For both **3a** and **3b**, the oxidative charge (16–17 µC·cm^−2^) was one-third of that observed for reductive desorption (Table 1), which was consistent with the 3:1 ratio of the densities of a S–Au bond and the bithiophene group, thus further confirming the achievement of three-point adsorption. The observed reversible behavior indicated that the phenyl end cap effectively improved the stability of the radical cation. In this context, the oxidized species from the parent bithiophene was reported to be stabilized by the capping with two terminal phenyl groups in solution [55]. The cyclic voltammetry of tris(thioacetate) **6b** in solution (Figure 7) also showed a reversible first oxidation wave (*E*_1/2_ = 0.740 V) and an irreversible second one (*E*_pa_ = 1.180 V). In the case of the SAM of **3b**, however, only the first oxidation wave was observed, because the oxidative desorption of a sulfur atom, RS–Au(s) + 2H_2_O → Au(s) + RSO_2_H + 3e^−^ + 3H^+^ [42], occurred at potentials higher than 1.1 V.

## 3. Experimental Section

### 3.1. General

Anhydrous solvents used for synthesis were prepared by standard methods. Other reagents were used as received, unless otherwise noted. Preparative gel permeation chromatography was performed in a recycle mode using Japan Analytical Industry JAIGEL 1H and 2H polystyrene columns, which were connected in a series.

^1^H-NMR spectra were obtained using either a JEOL AL400 (400 MHz) or a Varian Mercury-300 (300 MHz) instrument. ^13^C-NMR spectra were measured using a Varian Mercury 300 (75.5 MHz) instrument. High-resolution mass spectra were obtained using a JEOL JMS 700 spectrometer.

The IR spectra of neat samples were obtained with a Thermo-Mattson Infinity spectrometer using a drop-cast film prepared from a CHCl_3_ solution (5 mM) on a KBr plate at room temperature. The polarization modulation-infrared reflection absorption spectroscopy (PM-IRRAS) spectra were recorded using the same instrument equipped with a HgCdTe detector cooled with liquid N_2_ and a photoelastic modulator (Hinds, PEM-90) with an optical layout similar to that reported in the literature [56]. The signal was demodulated with a synchronous sampling demodulator (GWC Instruments). The differential reflectance was numerically converted to absorbance. All measurements were performed under an ambient atmosphere at room temperature.

### 3.2. Synthesis of 1-[4-(2,2′-Bithiophen-5-yl)phenyl]-3,5,7-tris(mercaptomethyl)adamantane (**3a**)

*5-(Tributylstannyl)-2,2′-bithiophene* (**5a**) [40]: To a solution of 2,2′-bithiophene (0.291 g, 1.75 mmol) in THF (25 mL) was added 1.25 mL of BuLi (1.54 M in hexane) over 5 min at −78 °C, and the mixture was stirred at this temperature for 30 min. Tributyltin chloride (0.69 g, 2.1 mmol) was added, and the mixture was stirred for 40 min at −78 °C and then for 12 h at room temperature. The mixture was quenched with saturated. NH_4_Cl, and the product was extracted with ether. The ether solution was dried (MgSO_4_) and evaporated, and the residue was purified by gel permeation chromatography (CHCl_3_) to give **5a** as a colorless oil (0.659 g, 83%): ^1^H-NMR (CDCl_3_, 300 MHz) δ 0.90 (t, *J* = 7.2 Hz, 9H), 1.09–1.14 (m, 6H), 1.35 (sext, *J* = 7.2 Hz, 6H), 1.56–1.61 (m, 6H), 6.98 (dd, *J* = 5.3, 3.5 Hz, 1H), 7.05 (d, *J* = 3.3 Hz, 1H), 7.14–7.17 (m, 2H), 7.28 (d, *J* = 3.3 Hz, 1H). ^13^C-NMR (CDCl_3_, 75.5 MHz) δ 10.9, 13.6, 27.2, 28.9, 123.4, 123.9, 124.9, 127.7, 136.0, 136.5, 137.7, 142.7.

*1-[4-(2,2′-Bithiophen-5-yl)phenyl]-3,5,7-tris(acetylthiomethyl)adamantane* (**6a**): THF (2.5 mL) was added to a mixture of tris(thioacetate) **4** [26] (45.9 mg, 0.0762 mmol), **5a** (59.1 mg, 0.130 mmol) and Pd(PPh_3_)_4_ (26.4 mg, 0.0228 mmol), and the mixture was stirred at 70 °C for 21 h under an argon atmosphere. The mixture was quenched with water, and the product was extracted with ether. The ether solution was washed with 10% NaCl and dried (MgSO_4_). The solvent was evaporated, and the residue was purified by gel permeation chromatography (CHCl_3_) to give **6a** as a colorless oil (13.6 mg, 28%): ^1^H-NMR (CDCl_3_, 400 MHz) δ 1.24 (d, *J* = 12.6 Hz, 3H), 1.28 (d, *J* = 11.9 Hz, 3H), 1.55 (s, 6H), 2.36 (s, 9H), 2.86 (s, 6H), 7.03 (dd, *J* = 5.1, 3.6 Hz, 1H), 7.13 (d, *J* = 3.9 Hz, 1H), 7.18–7.20 (m, *2*H), 7.21 (dd, *J* = 5.1, 1.0 Hz, 1H), 7.30 (d, *J* = 8.5 Hz, 2H), 7.54 (d, *J* = 8.5 Hz, 2H). ^13^C-NMR (CDCl_3_, 75.5 MHz) δ 30.7, 35.8, 38.3, 41.0, 43.7, 45.4, 123.48, 123.53, 124.3, 124.5, 125.49, 125.53, 127.8, 131.9, 136.4, 137.4, 142.8, 148.0, 195.3. HRMS (EI+) *m/z* calcd. for C_33_H_36_O_3_S_5_ (M^+^) 640.1268, found 640.1262.

*1-[4-(2,2′-Bithiophen-5-yl)phenyl]-3,5,7-tris(mercaptomethyl)adamantane* (**3a**): A solution of tris(thioacetate) **6a** (8.2 mg, 0.013 mmol) in THF (3.5 mL) was added to a stirred suspension of LiAlH_4_ (25.6 mg, 0.675 mmol) in THF (1 mL) over a period of 5 min. The reaction mixture was stirred at 0 °C for 1 h and quenched by the addition of 10% HCl (1.5 mL). The product was extracted with CH_2_Cl_2_, and the organic layer was washed with 10% NaCl and dried (MgSO_4_). The solvent was evaporated to give **3a** as a colorless oil (6.2 mg, 94%): ^1^H-NMR (CDCl_3_, 400 MHz) δ 1.21 (t, *J* = 8.8 Hz, 3H), 1.31 (d, *J* = 12.4 Hz, 3H), 1.35 (d, *J* = 12.1 Hz, 3H), 1.61 (s, 6H), 2.49 (d, *J* = 9.0 Hz, 6H), 7.03 (dd, *J* = 5.1, 3.6 Hz, 1H), 7.14 (d, *J* = 3.9 Hz, 1H), 7.19–7.23 (m, 3H), 7.38 (d, *J* = 8.5 Hz, 2H), 7.56 (d, *J* = 8.5 Hz, 2H). ^13^C-NMR (CDCl_3_, 75.5 MHz) δ 35.9, 37.5, 38.4, 43.1, 45.4, 123.49, 123.52, 124.3, 124.5, 125.5, 125.6, 127.8, 131.9, 136.4, 137.4, 142.8, 148.4. HRMS (EI+) *m/z* calcd. for C_27_H_30_S_5_ (M^+^) 514.0951, found 514.0944.

### 3.3. Synthesis of 1-[4-(5′-Phenyl-2,2′-bithiophen-5-yl)phenyl]-3,5,7-tris(mercaptomethyl)adamantane (**3b**)

*5-Phenyl-2,2′-bithiophene* [57]: A DMF solution (2.5 mL) of bromobenzene (105 mg, 0.67 mmol) was added to a mixture of Pd(PPh_3_)_4_ (22.4 mg, 0.0194 mmol) and CuO (28.4 mg, 0.357 mmol) under an argon atmosphere. A DMF solution (1 mL) of **5a** (153 mg, 0.336 mmol) was added at 100 °C, and the mixture was stirred at 100 °C for 1 h, filtered through a short SiO_2_ column and evaporated. The residue was purified by gel permeation chromatography (CHCl_3_) to give 5-phenyl-2,2*′*-bithiophene as a yellow solid (71.5 mg, 88%). ^1^H-NMR (CDCl_3_, 300 MHz) δ 7.01 (dd, *J* = 5.2, 3.6 Hz, 1H), 7.12 (d, *J* = 3.6 Hz, 1H), 7.16–7.21 (m, 3H), 7.27 (d, *J* = 7.5 Hz, 1H), 7.36 (t, *J* = 7.8 Hz, 2H), 7.58 (d, *J* = 7.8 Hz, 2H). ^13^C-NMR (CDCl_3_, 75.5 MHz) δ 123.6, 123.7, 124.3, 124.5, 125.5, 127.5, 127.8, 128.9, 134.0, 136.6, 137.4, 143.0.

*5-(Tributylstannyl)-5′-phenyl-2,2′-bithiophene* (**5b**): To a solution of 5-phenyl-2,2′-bithiophene (71.5 mg, 0.295 mmol) in THF (5 mL) was added 0.22 mL of BuLi (1.6 M in hexane) over 5 min at −78 °C, and the mixture was stirred at this temperature for 30 min. Tributyltin chloride (0.17 g, 0.52 mmol) was added, and the mixture was stirred for 40 min at −78 °C and for 19 h at room temperature. The mixture was quenched with sat. NH_4_Cl, and the product was extracted with ether. The ether solution was dried (MgSO_4_) and evaporated, and the residue was purified by gel permeation chromatography (CHCl_3_) to give **5b** as a yellow oil (0.147 g, 94%): ^1^H-NMR (CDCl_3_, 300 MHz) δ 0.91 (t, *J* = 7.4 Hz, 9H), 1.10–1.15 (m, 6H), 1.35 (sext, *J* = 7.3 Hz, 6H), 1.54–1.62 (m, 6H), 7.07 (d, *J* = 3.3 Hz, 1H), 7.13 (d, *J* = 3.6 Hz, 1H), 7.21 (d, *J* = 3.9 Hz, 1H), 7.27 (d, *J* = 7.5 Hz, 1H), 7.31 (d, *J* = 3.3 Hz, 1H), 7.36 (t, *J* = 7.5 Hz, 2H), 7.59 (d, *J* = 7.5 Hz, 2H). ^13^C-NMR (CDCl_3_, 75.5 MHz) δ 10.9, 13.7, 27.3, 28.9, 123.7, 124.3, 124.8, 125.5, 127.4, 128.9, 134.2, 136.1, 136.8, 137.0, 142.6, 142.7.

*1-[4-(5′-Phenyl-2,2′-bithiophen-5-yl)phenyl]-3,5,7-tris(acetylthiomethyl)adamantane* (**6b**): THF (4.5 mL) was added to a mixture of tris(thioacetate) **4** [26] (77.9 mg, 0.129 mmol), **5b** (86.2 mg, 0.162 mmol) and Pd(PPh_3_)_4_ (30.0 mg, 0.0260 mmol), and the mixture was stirred at 70 °C for 22 h under an argon atmosphere. The mixture was quenched with water, and the product was extracted with ether. The ether solution was washed with 10% NaCl and dried (MgSO_4_). The solvent was evaporated, and the residue was purified by gel permeation chromatography (CHCl_3_) to give **6b** as a yellow solid (20.6 mg, 22%): ^1^H-NMR (CDCl_3_, 300 MHz) δ 1.23 (d, *J* = 12.6 Hz, 3H), 1.28 (d, *J* = 12.3 Hz, 3H), 1.55 (s, 6H), 2.36 (s, 9H), 2.86 (s, 6H), 7.15 (d, *J* = 3.6 Hz, 1H), 7.16 (d, *J* = 3.9 Hz, 1H), 7.20 (d, *J* = 3.9 Hz, 1H), 7.24 (d, *J* = 3.6 Hz, 1H), 7.28–7.32 (m, 3H), 7.39 (d, *J* = 7.5 Hz, 2H), 7.54 (d, *J* = 8.4 Hz, 2H), 7.61 (d, *J* = 8.4 Hz, 2H). ^13^C-NMR (CDCl_3_, 75.5 MHz) δ 30.7, 35.8, 38.3, 41.0, 43.7, 45.4, 123.6, 123.8, 124.40, 124.45, 125.52, 125.55, 125.57, 127.6, 128.9, 131.9, 134.0, 136.4, 136.7, 142.9, 143.0, 148.1, 195.3. HRMS (EI+) *m/z* calcd. for C_39_H_40_O_3_S_5_ (M^+^) 716.1581, found 716.1547.

*1-[4-(5′-Phenyl-2,2′-bithiophen-5-yl)phenyl]-3,5,7-tris(mercaptomethyl)adamantane* (**3b**): A solution of tris(thioacetate) **6b** (20.6 mg, 0.0287 mmol) in THF (6 mL) was added to a stirred suspension of LiAlH_4_ (86.5 mg, 2.28 mmol) in THF (2.5 mL) over a period of 10 min. The reaction mixture was stirred at 0 °C for 1 h and quenched by the addition of 10% HCl (2 mL). The product was extracted with CH_2_Cl_2_, and the organic layer was washed with water and dried (MgSO_4_). The solvent was evaporated, and the residue was purified by flash column chromatography on silica gel (CH_2_Cl_2_) to give **3b** as a yellow solid (14.7 mg, 87%): ^1^H-NMR (CDCl_3_, 300 MHz) δ 1.21 (t, *J* = 8.7 Hz, 3H), 1.32 (s, 6H), 1.61 (s, 6H), 2.49 (d, *J* = 9.0 Hz, 6H), 7.16 (d, *J* = 3.9 Hz, 1H), 7.17 (d, *J* = 3.9 Hz, 1H), 7.21 (d, *J* = 3.6 Hz, 1H), 7.24 (d, *J* = 3.9 Hz, 1H), 7.29–7.41 (m, 5H), 7.56 (d, *J* = 8.7 Hz, 2H), 7.61 (d, *J* = 8.4 Hz, 2H). ^13^C-NMR (CDCl_3_, 75.5 MHz) δ 35.9, 37.5, 38.5, 43.1, 45.5, 123.6, 123.8, 124.39, 124.44, 125.51, 125.55, 125.58, 127.6, 128.9, 131.9, 134.0, 136.4, 136.7, 142.8, 143.0, 148.4. HRMS (FAB+) *m/z* calcd. for C_33_H_34_S_5_ (M^+^) 590.1264, found 590.1273.

### 3.4. Preparation of the Self-Assembled Monolayer on a Au(111) Substrate

Gold (99.99%) was vapor-deposited on freshly cleaved mica sheets (0.05 mm thickness, 6 cm × 6 cm). The mica was pre-baked at 580 °C under high vacuum (<10^−3^ Pa) for at least 6 h. The deposition was carried out at this temperature with an evaporation rate of 1.0–1.5 nm·s^−1^ until a gold thickness of 200 ± 5 nm was reached. The obtained substrate was cut into 1 cm × 2 cm pieces and annealed at 530 °C in a furnace for 8 h under air to remove the surface contamination and to minimize defects. The hot pieces were quenched in Millipore purified water (resistivity >18 MΩ·cm, Millipore Simplicity 185 Water System). STM analysis of the gold film, thus prepared, showed the formation of large Au(111) terraces. The substrate was consecutively rinsed with EtOH and AcOEt and immersed in a 0.1 mM solution of **3a** in AcOEt. After 24 h, or longer, in the dark, the substrate was rinsed with pure AcOEt and dried under an ambient atmosphere. The SAM of **3b** was prepared in a similar manner using CH_2_Cl_2_ instead of AcOEt.

### 3.5. Cyclic Voltammetry

A surface-modified gold substrate was mounted at the bottom of a cone-shaped cell using an O-ring and a clamp to serve as a working electrode. The area of the electrode exposed to the electrolyte was 0.152 cm^2^ (4.4 mm diameter circle). Reductive desorption was recorded with aqueous 0.5 M KOH using a Ag/AgCl/sat. KCl reference electrode and a platinum wire counter electrode. Oxidative scans were conducted in a CH_2_Cl_2_ solution containing 0.1 M tetrabutylammonium perchlorate (TBAP) using a Ag/AgNO_3_ (0.01 M in CH_3_CN) reference electrode and a platinum wire counter electrode. A Luggin capillary (0.5 mm bore) was placed at a distance of about 1 mm from the surface of the working electrode in order to minimize the IR drop in the solution. The electrolyte solution in the cell was deaerated by bubbling argon for 10 min before scanning. Voltammograms were recorded on a BAS 100B electrochemical analyzer.

### 3.6. Theoretical Calculations

Density functional theory (DFT) calculations [58] were performed using the Gaussian 03 program [59]. Geometry optimization was carried out at the B3LYP/6-31G(d) level. The resultant geometries were verified by frequency calculations to have no imaginary frequencies.

## 4. Conclusions

Dyads **3a** and **3b**, prepared from bithiophene and adamantane tripodal trithiol, formed SAMs on Au(111) via three-point adsorption, as evidenced by PM-IRRAS measurements and the charge of reductive desorption. A reversible cyclic voltammogram for the single-electron oxidation was obtained by capping the terminal thiophene ring with a phenyl group. An increase in the two-dimensional bulkiness due to the possible *anti*-*syn* conformational fluctuation resulted in surface coverage that was lower than that observed for a ferrocene-terminated analogue **2**. This is in contrast to the fact that *n*-alkanethiols form closely packed SAMs, in which flexible alkyl chains are fixed at a linear conformation to maximize the intermolecular affinity. The adamantane tripod permits self-assembly in a well-controlled manner and serves as a rigid junction between a device molecule and a metal electrode. The present results provides insight into the molecular geometry of oligothiophene SAMs, which are promising systems for organic optoelectronic devices.

## Supplementary Materials

Supplementary materials can be accessed at http://www.mdpi.com/1420-3049/19/9/15298/s1.

## Supplementary Files

Supplementary File 1
