# Supplementary material for: Electrochemistry of the Self-Assembled Monolayers of Dyads Consisting of Tripod-Shaped Trithiol and Bithiophene on Gold
Source: Molecules. 2014 Sep 24;19(9):15298–313. doi: 10.3390/molecules190915298 (PMC6271350; doi:10.3390/molecules190915298)
Supplement: Supplementary File 1 [file molecules-19-15298-s001.pdf]

Supplementary Materials

Figure S1. ^{1}H-NMR spectrum of 5-phenyl-2,2′-bithiophene (300 MHz, $CDCl_3$).

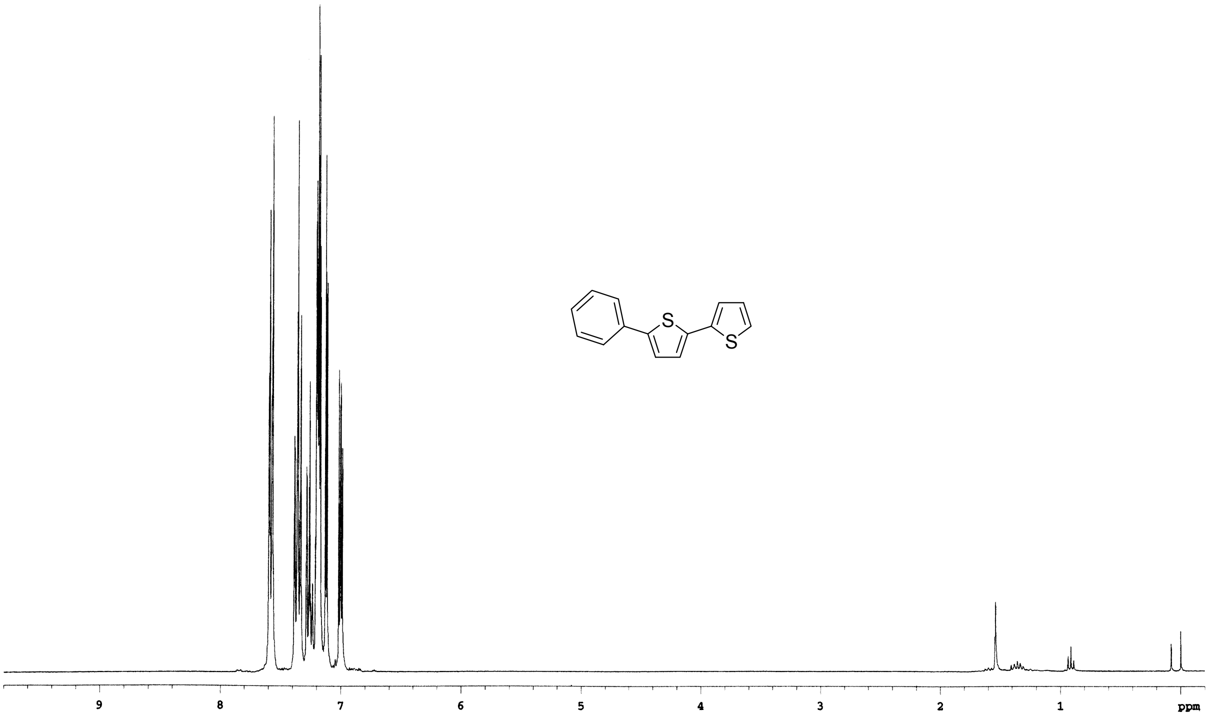

Figure S2. ^{13}C-NMR spectrum of 5-phenyl-2,2′-bithiophene (75.5 MHz, $CDCl_3$).

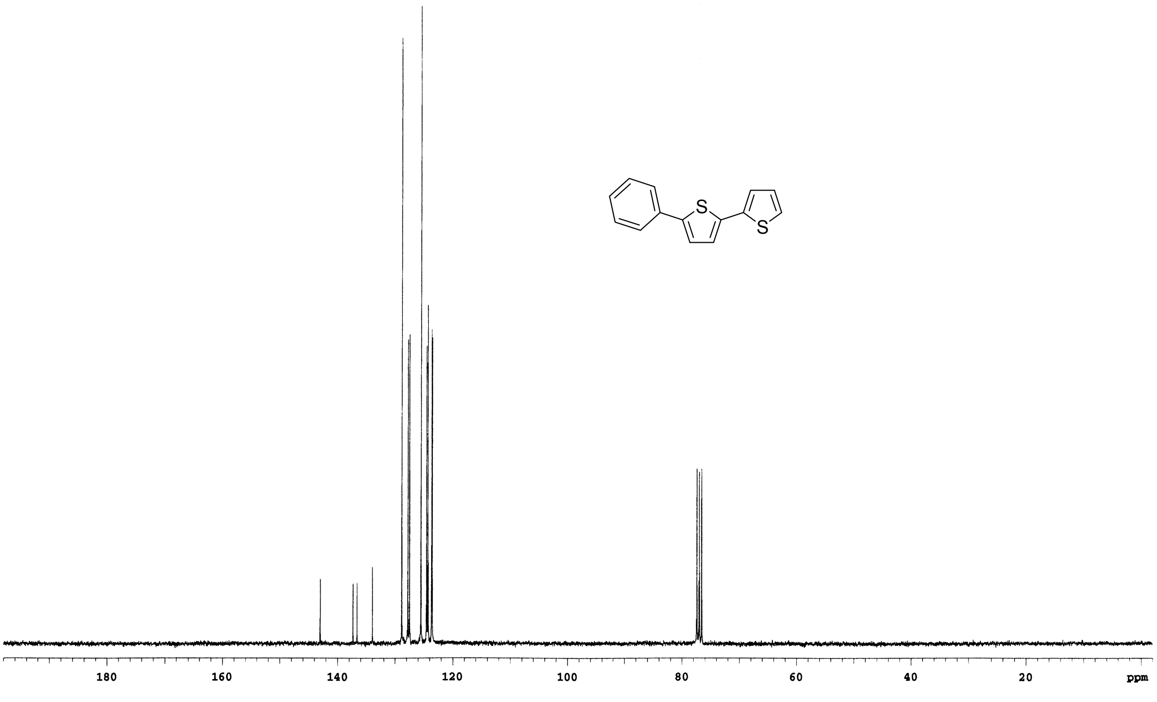

Figure S3. ^{1}H-NMR spectrum of **3a** (400 MHz, $CDCl_3$).

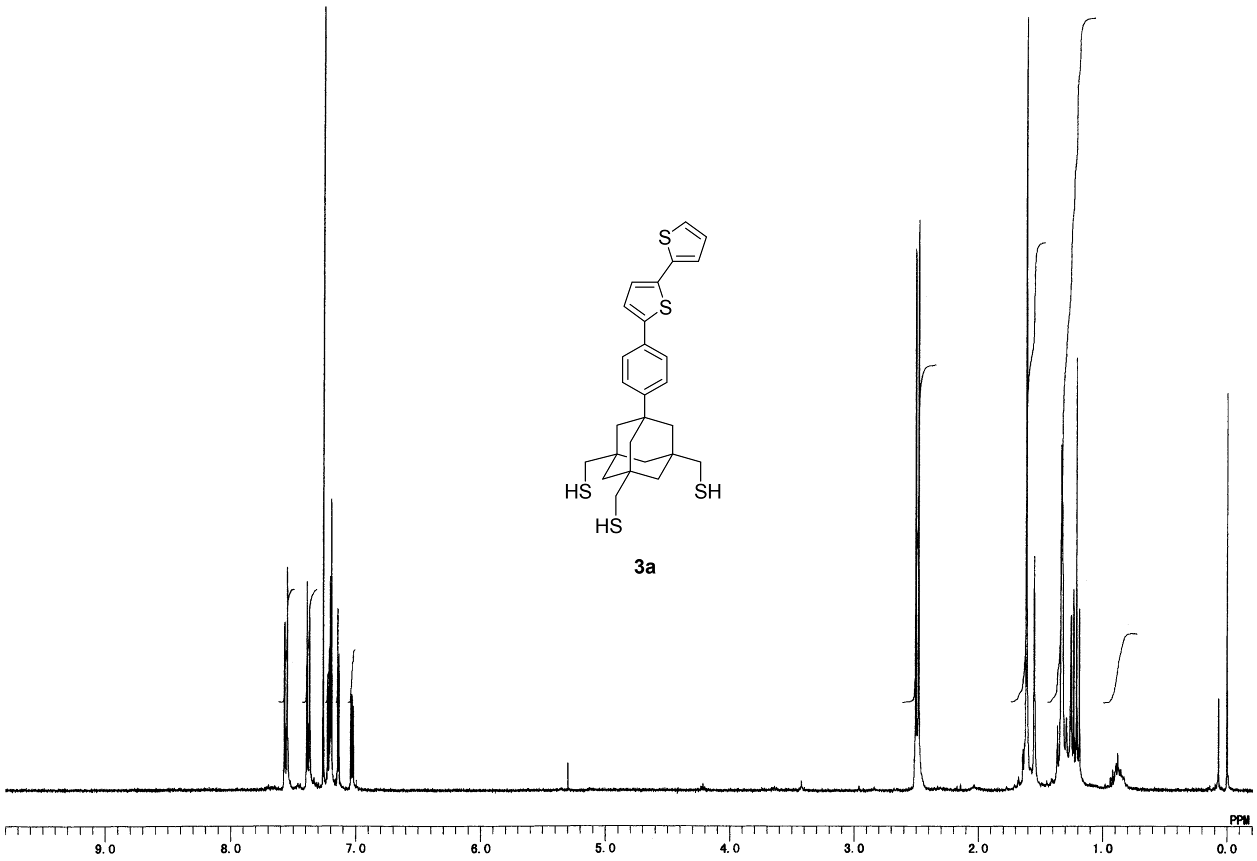

Figure S4. ^{13}C-NMR spectrum of **3a** (75.5 MHz, $CDCl_3$).

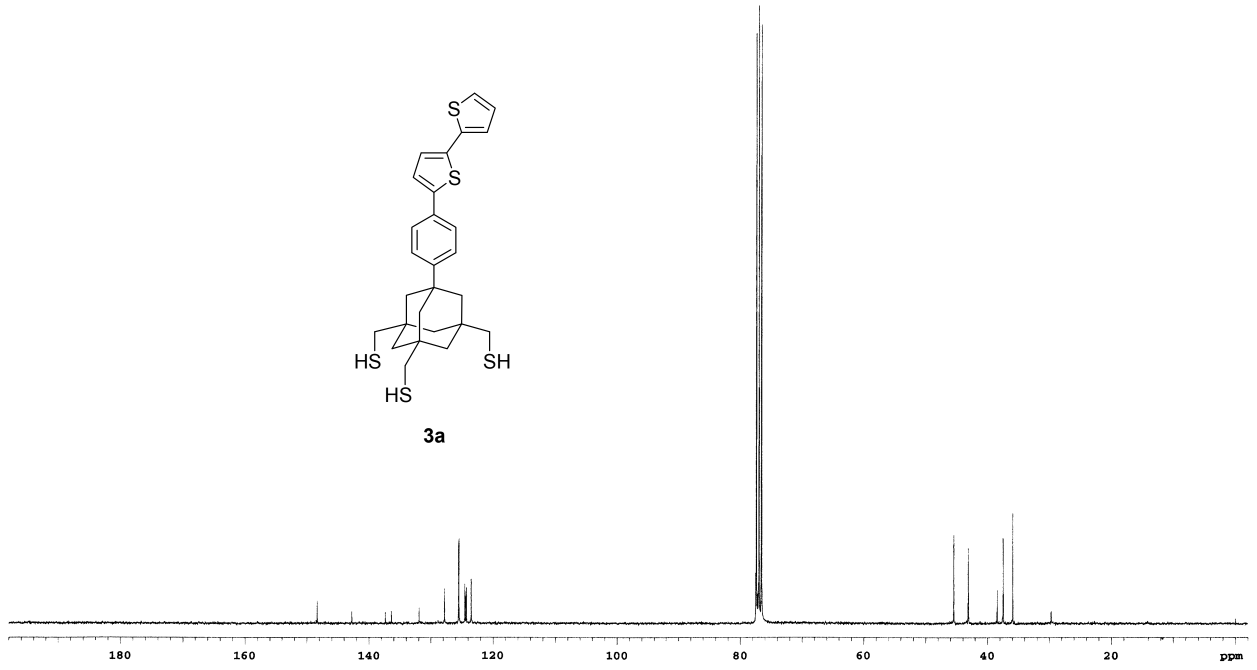

Figure S5. ^{1}H-NMR spectrum of **3b** (300 MHz, $CDCl_3$).

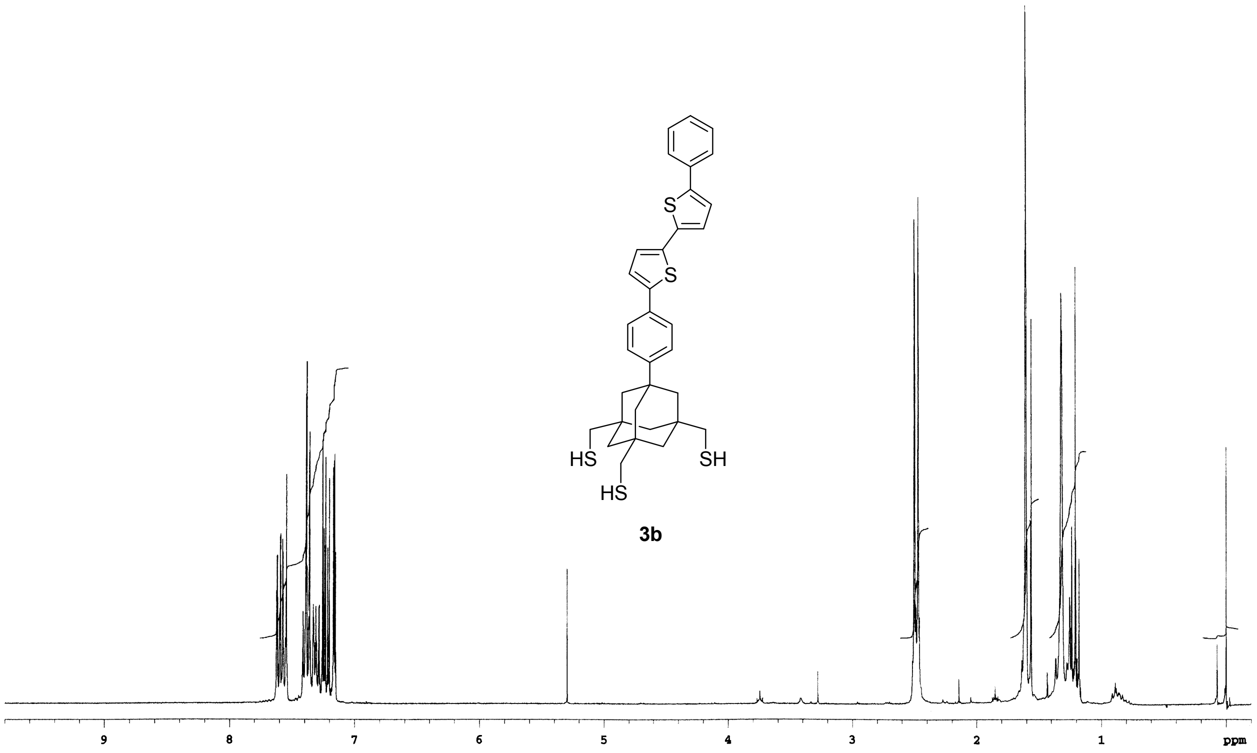

Figure S6. ^{13}C-NMR spectrum of **3b** (75.5 MHz, $CDCl_3$).

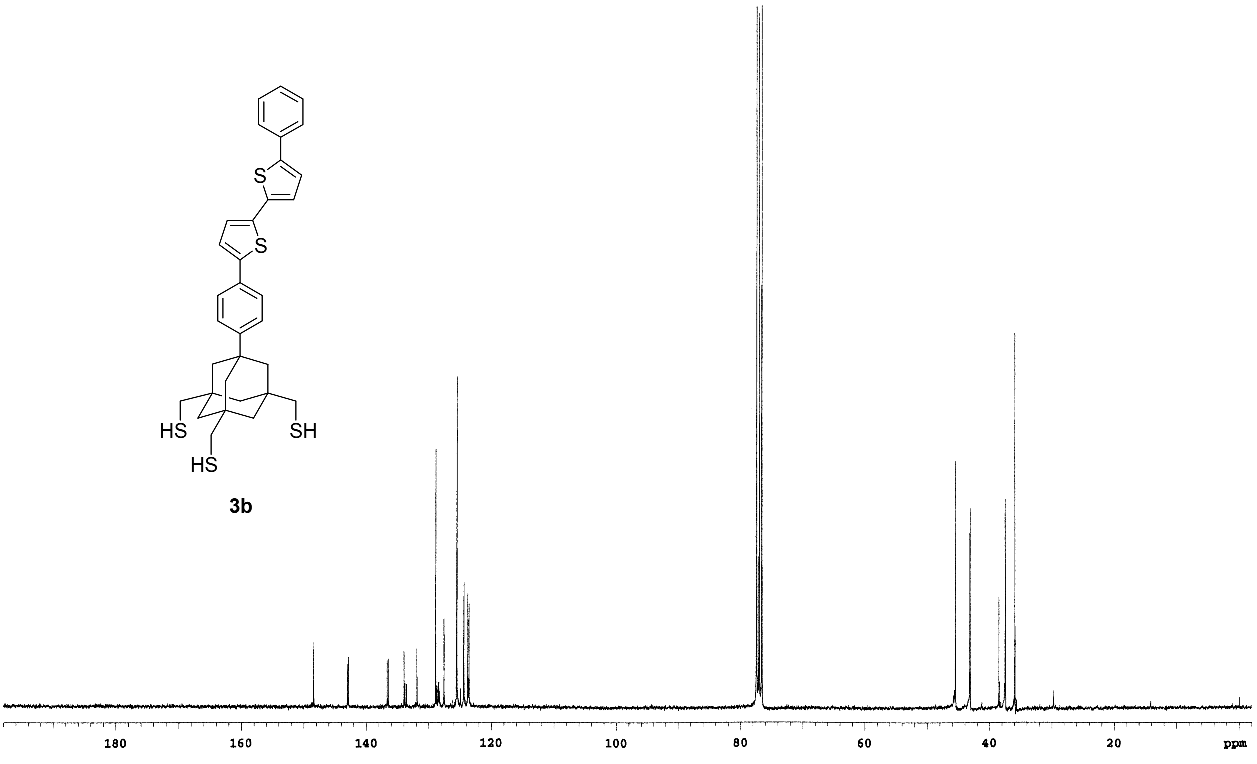

Figure S7. ^{1}H-NMR spectrum of **5a** (300 MHz, $CDCl_3$).

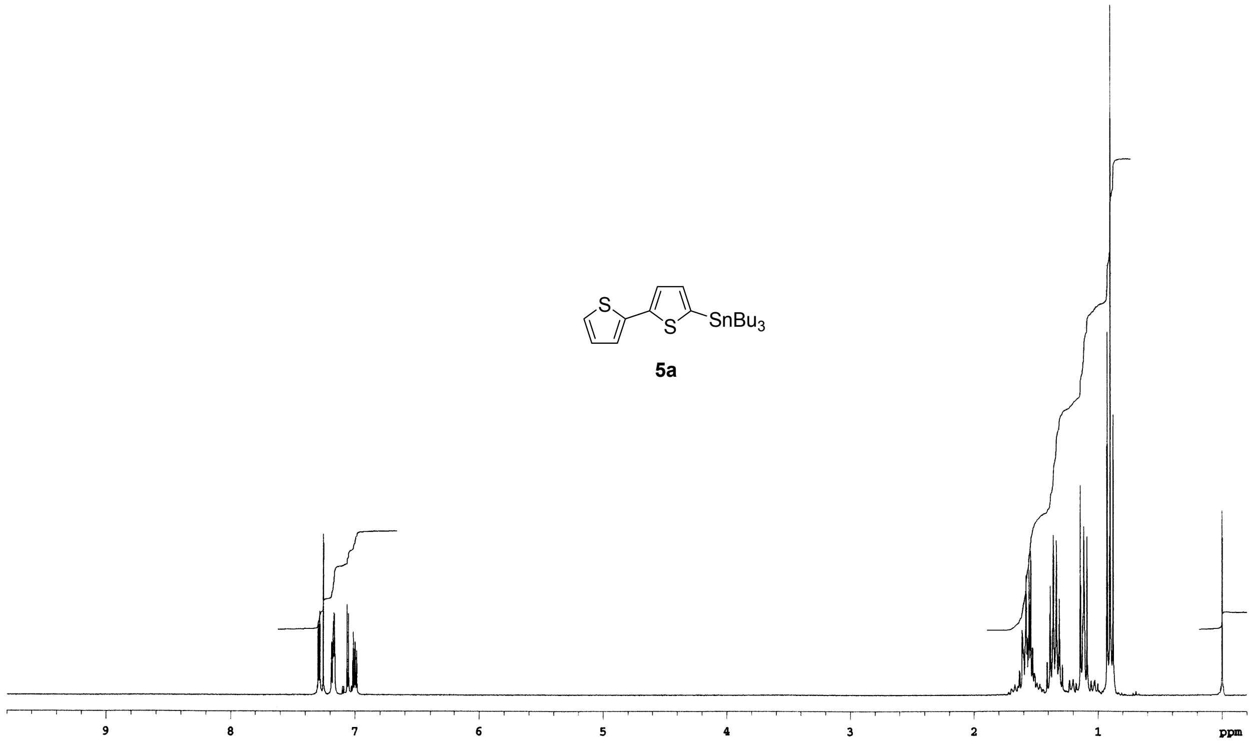

Figure S8. ^{13}C-NMR spectrum of **5a** (75.5 MHz, $CDCl_3$).

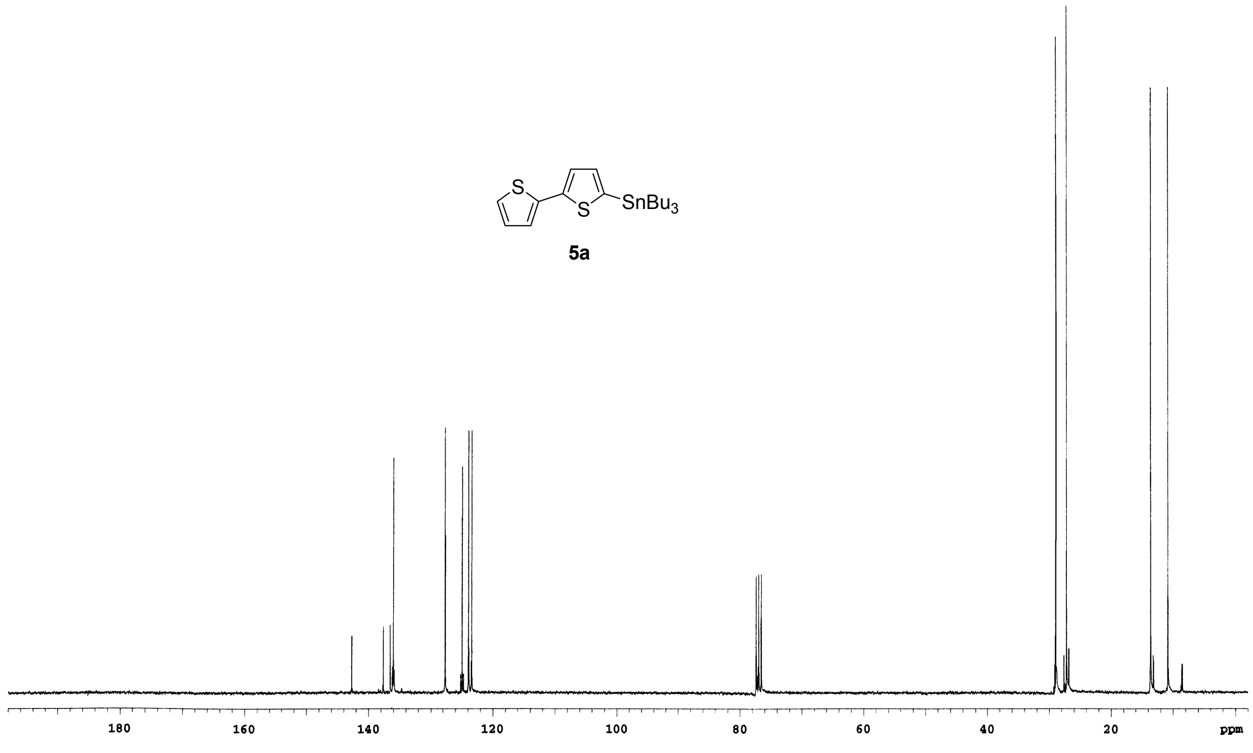

Figure S9. ^{1}H-NMR spectrum of **5b** (300 MHz, $CDCl_3$).

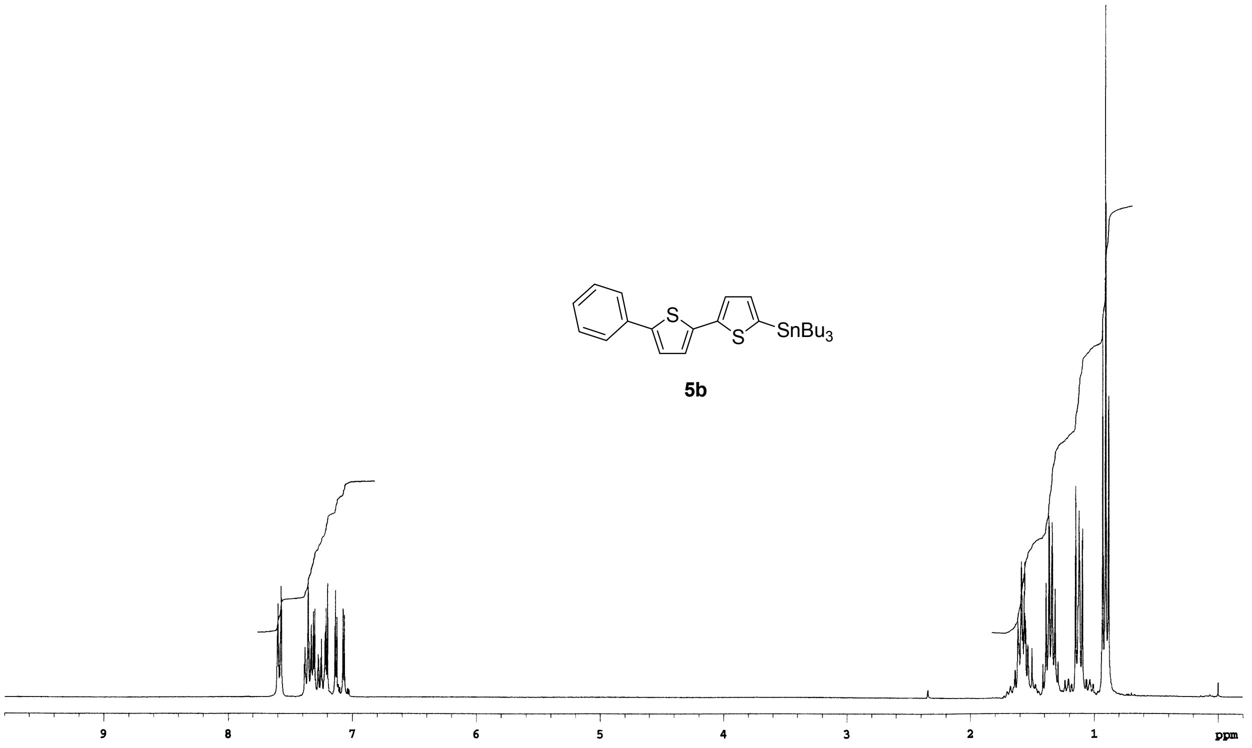

Figure S10. ^{13}C-NMR spectrum of **5b** (75.5 MHz, $CDCl_3$).

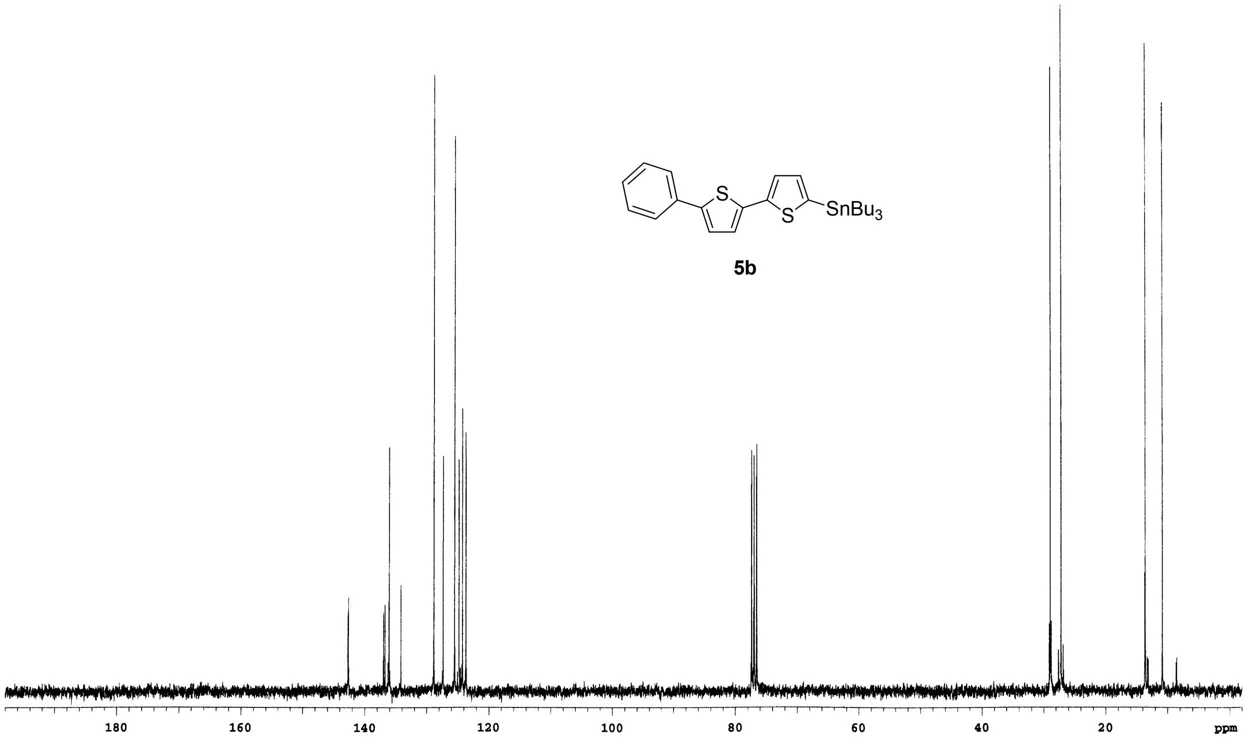

Figure S11. ^{1}H-NMR spectrum of **6a** (400 MHz, $CDCl_3$).

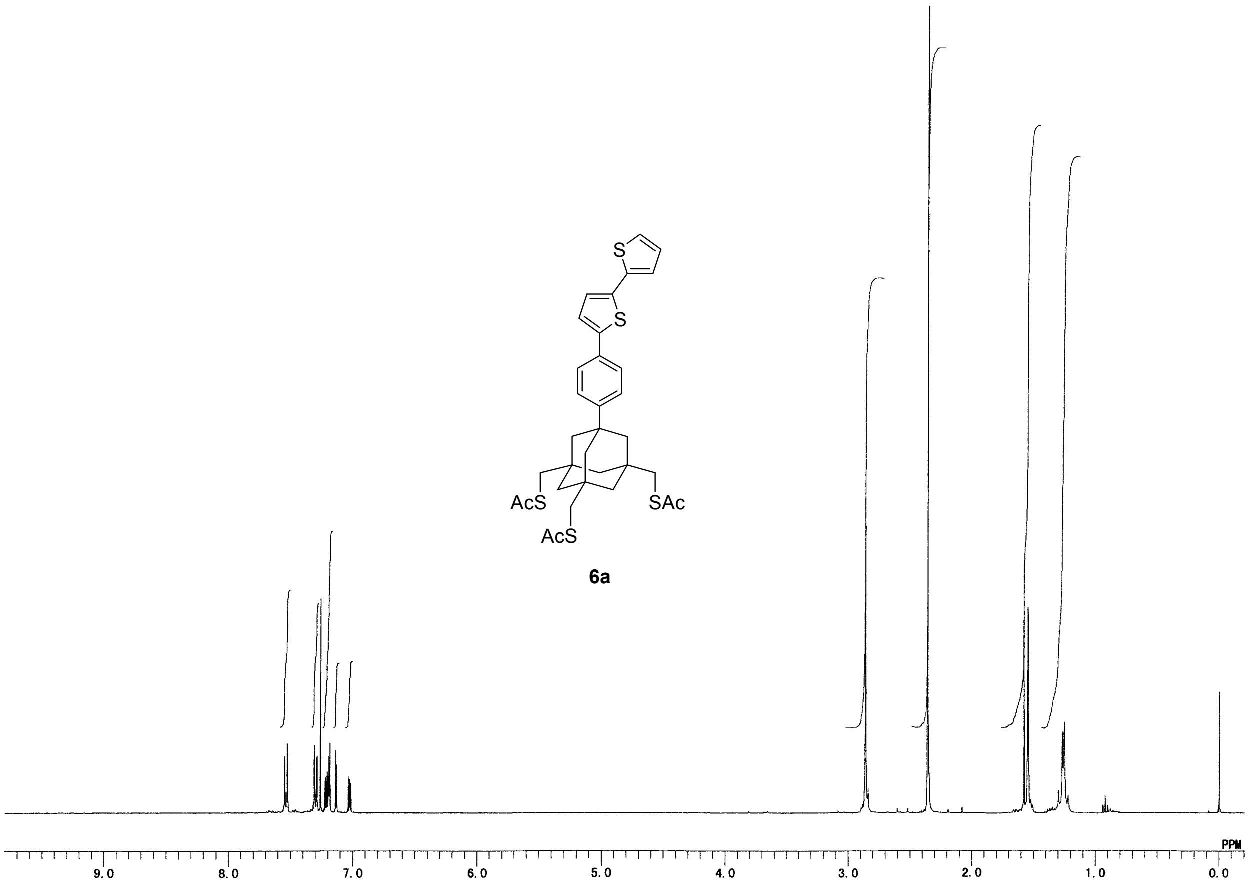

Figure S12. ^{13}C-NMR spectrum of **6a** (75.5 MHz, $CDCl_3$).

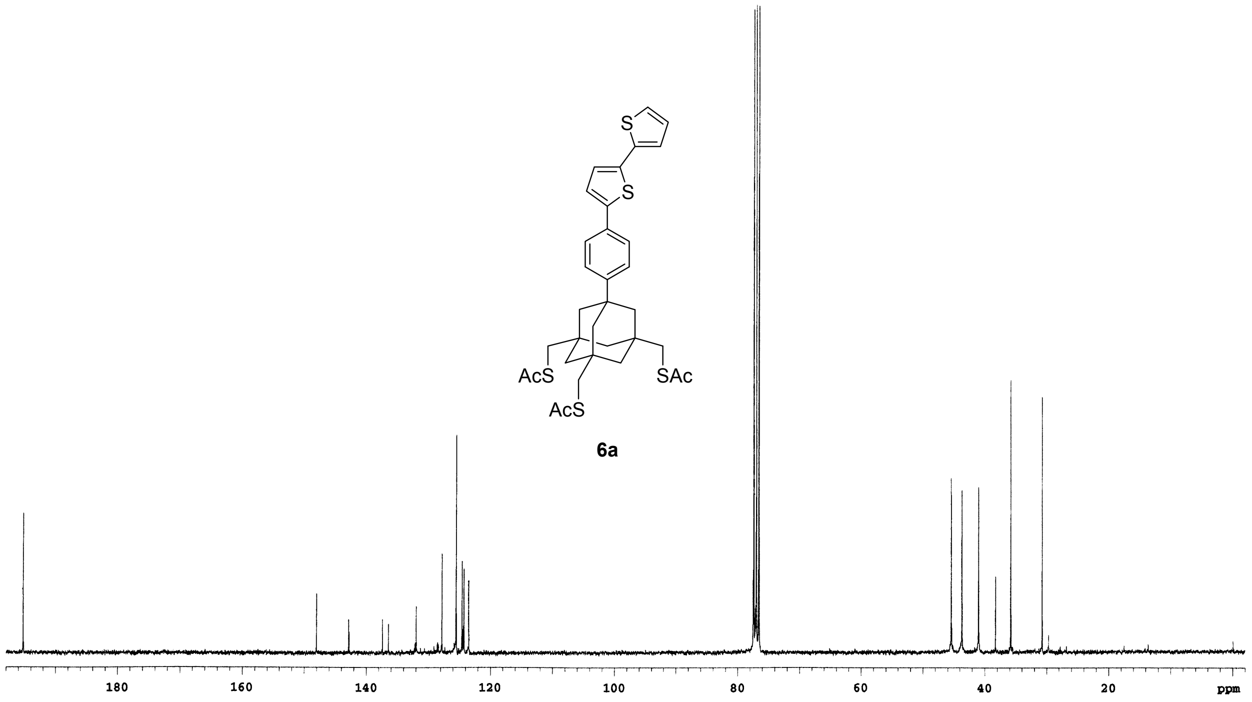

Figure S13. ^{1}H-NMR spectrum of **6b** (300 MHz, $CDCl_3$).

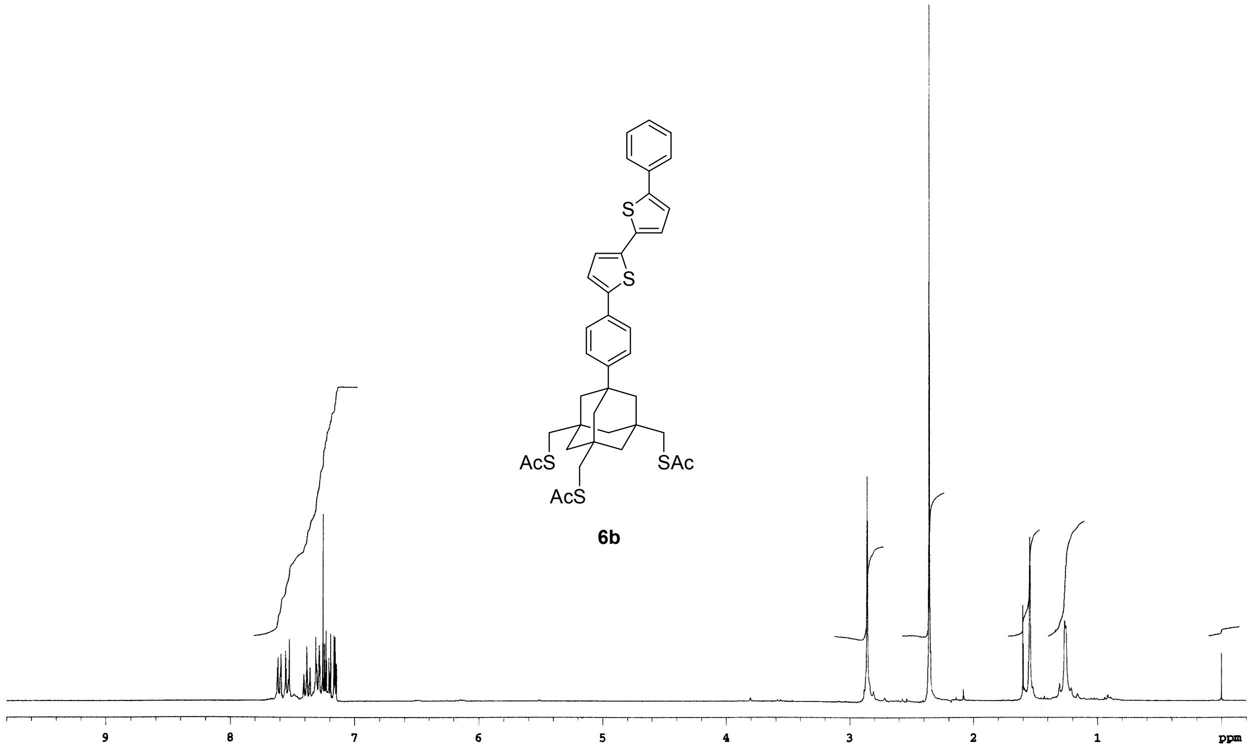

Figure S14. ^{13}C-NMR spectrum of **6b** (75.5 MHz, $CDCl_3$).

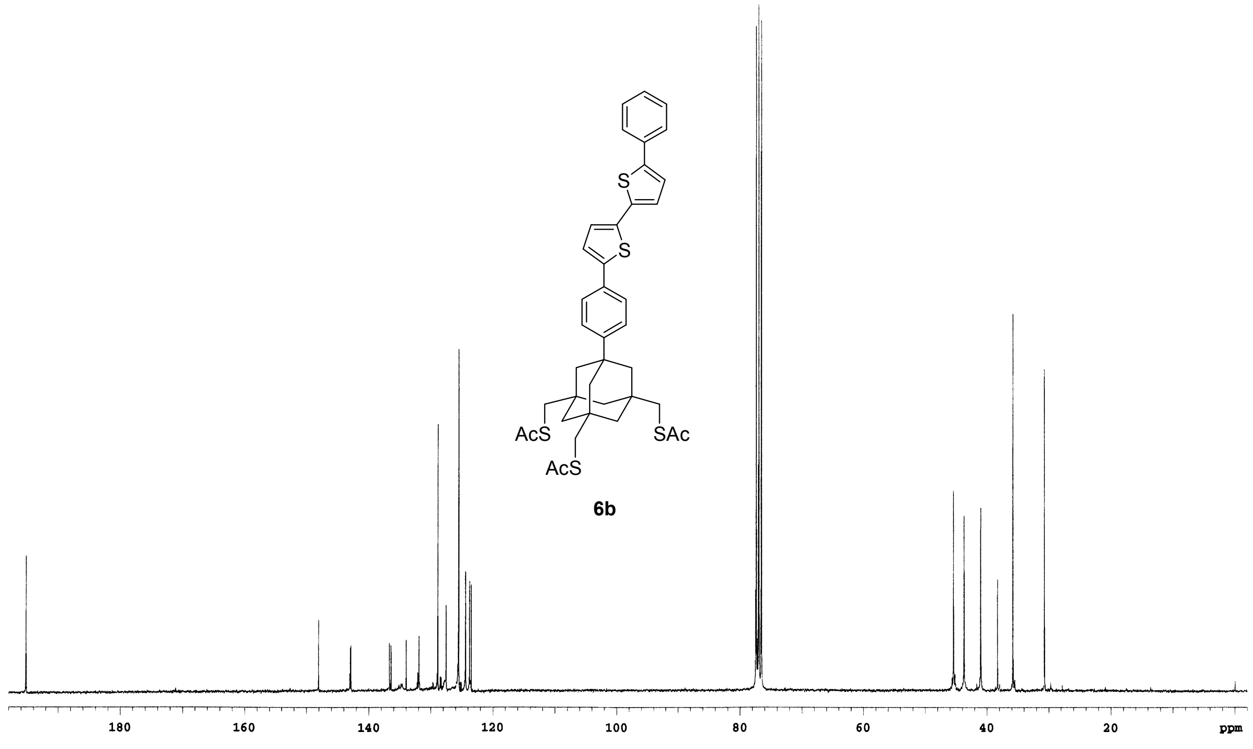

Table S1. Cartesian coordinates and energies for the DFT-optimized structure of 3b (*anti* form).

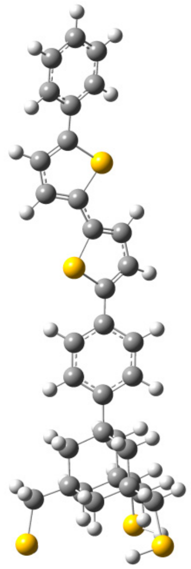

Level of theory: B3LYP/6-31G(d); Number of imaginary frequencies: 0; Total Electronic Energy (E): −3268.9522052 hartree; Zero Point Energy (ZPE): 0.583039 hartree; Gibbs Free Energy (G): −3268.439440 hartree.

Atom	X	Y	Z
C	−5.5527776864	1.4508630251	−0.4856098308
C	−6.0256201796	1.2306208374	0.9669003242
C	−5.5863967507	−0.1546721045	1.4833524364
C	−6.1782431268	−1.2439814097	0.5630436764
C	−5.7145937966	−1.0522527404	−0.897719044
C	−6.1524506408	0.3460034618	−1.3807588207
C	−4.0423627264	−0.2371409773	1.4172446326
C	−4.0089193009	1.3384945509	−0.5285127436
C	−4.1692682285	−1.1267206	−0.9382633546
C	−5.921006898	2.8585648026	−0.9989710386
S	−7.7053485058	3.3343135283	−1.0125997717
C	−5.9946028178	−0.3668341818	2.9560375958
S	−7.7972274516	−0.3874405611	3.3594134087
C	−6.2525981469	−2.1685663404	−1.8162214898
S	−8.081684597	−2.3030067158	−2.0346790087
C	−3.5215601967	−0.042821413	−0.0335105286
C	2.29826186	−0.7085322486	−0.1326725015
C	0.2424846454	0.6740578553	−0.5451801882
C	−1.1419222177	0.8360081329	−0.519015815
C	−1.9940788582	−0.1805633693	−0.0665638496
C	−1.3828820842	−1.3707784253	0.3684700159
C	−0.0040882602	−1.5357663452	0.357457692
C	0.8467445197	−0.5151292503	−0.1084055547
H	−5.6080251149	2.0165518058	1.614899125
H	−7.1160416455	1.3241439543	1.010129826
H	−7.2714981102	−1.208092766	0.6201955859
H	−5.868973046	−2.237713145	0.922443131

Table S1. *Cont.*

Atom	X	Y	Z
H	−7.2460723268	0.4059622026	−1.3740661273
H	−5.826668162	0.4949355268	−2.421732344
H	−3.7124996458	−1.208913251	1.806159784
H	−3.5949206909	0.5281950782	2.066297787
H	−3.6627230672	1.5075272625	−1.5573844555
H	−3.5697867872	2.1317618696	0.092073034
H	−3.8127201823	−0.9954566369	−1.9694580967
H	−3.8408350396	−2.1243831738	−0.6191843826
H	−5.4580133479	3.6159350776	−0.3560482863
H	−5.5192268649	3.0080219654	−2.006938992
H	−8.088829366	2.5560953834	−2.0466501777
H	−5.5104972005	0.3820112833	3.5920846972
H	−5.6442762275	−1.348011832	3.2962317234
H	−8.0346358666	0.9286063483	3.1761788474
H	−5.8755481434	−3.1424804203	−1.4860341514
H	−5.882563465	−2.0131700946	−2.8360318065
H	−8.3715466934	−2.7859589473	−0.8081377466
H	0.8592334222	1.4814882656	−0.9310834859
H	−1.5504731951	1.7767890883	−0.870809261
H	−1.9939541742	−2.1895085898	0.7385594144
H	0.423490081	−2.4604546348	0.7337828269
S	3.4061267813	0.6533064642	−0.1036797278
C	3.0078501711	−1.8895833266	−0.1720885082
C	4.8112294806	−0.395906988	−0.1812639472
H	2.5289838894	−2.8614687245	−0.2194433936
C	4.414046377	−1.7173018339	−0.2002624811
C	6.1474375416	0.1554816542	−0.2095879059
H	5.1174438168	−2.54085158	−0.264390641
C	6.554900508	1.4351009652	−0.5270064682
S	7.5374445634	−0.8333502174	0.203259638
H	5.8628331162	2.2129762458	−0.8322384427
C	7.9584602155	1.6189087579	−0.4586545598
C	8.6549522029	0.4880305066	−0.0909260506
H	8.448015258	2.552348313	−0.7138395953
C	10.1023451267	0.3165693753	0.0679934734
C	10.7186099647	−0.9399576885	−0.0774351403
C	10.9172345619	1.4248436231	0.3691087154
H	10.4604143019	2.398777278	0.5178145063
C	12.2953117675	1.2815548844	0.5053701622
H	10.1151514443	−1.8078640199	−0.3294473464
C	12.0964340474	−1.0819976886	0.0711339768
H	12.5494551932	−2.0625721274	−0.0481606222
C	12.8931276554	0.0275259659	0.3591521452
H	12.9035536215	2.1513100114	0.7393292878
H	13.9679578046	−0.0836924182	0.4717687689

Table S2. Cartesian coordinates and energies for the DFT-optimized structure of 3b (*syn* form).

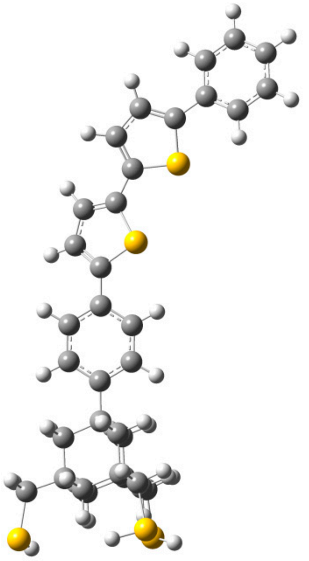

Level of theory: B3LYP/6-31G(d); Number of imaginary frequencies: 0; Total Electronic Energy (E): −3268.9508618 hartree; Zero Point Energy (ZPE): 0.582656 hartree; Gibbs Free Energy (G): −3268.438873 hartree.

Atom	X	Y	Z
C	−5.8058370746	−1.1119778611	−0.2354260584
C	−6.0226783557	−0.0816775024	−1.3632243214
C	−5.1365303005	1.1632817423	−1.1486390775
C	−5.4829234852	1.7940847084	0.2176336648
C	−5.270191042	0.7857615052	1.3675392841
C	−6.152726309	−0.4541812739	1.1166881502
C	−3.6544685834	0.7164549737	−1.1233179486
C	−4.3131278745	−1.5230908525	−0.219043886
C	−3.7867620021	0.3429048554	1.3659973203
C	−6.6299587228	−2.3951100486	−0.4696985654
S	−8.4642682902	−2.2308155904	−0.6067877814
C	−5.2903687975	2.1778271721	−2.3003205032
S	−6.9444624047	2.9615793829	−2.5480294674
C	−5.5570964197	1.422199913	2.7432512808
S	−7.2787516176	1.9938105225	3.0907900151
C	−3.3862857526	−0.3072091474	0.0133074164
C	2.3088624446	−1.6809863186	0.1000269224
C	−0.0884964546	−2.3160734602	−0.1650872497
C	−1.4457503851	−2.0019441339	−0.1757097989
C	−1.9010632628	−0.6915888913	0.031397533
C	−0.9200962695	0.2902481484	0.2545034031
C	0.4350003191	−0.0167582372	0.2803311861
C	0.8862175654	−1.3327319011	0.0710661437
H	−5.785255634	−0.5419276009	−2.3347275482
H	−7.0794932652	0.2048979062	−1.3930527333
H	−6.5234762318	2.1359493669	0.2021827338
H	−4.8541848124	2.6824946713	0.383614549
H	−7.2072902428	−0.1589753263	1.133695366

Table S2. *Cont.*

Atom	X	Y	Z
H	−6.0075480454	−1.1806412867	1.9310545085
H	−3.0100251838	1.5955158817	−0.9924638176
H	−3.3778620977	0.2664898516	−2.0868439807
H	−4.1502657886	−2.2708678665	0.5692974467
H	−4.0621470462	−2.0044245964	−1.1742751901
H	−3.6069062704	−0.3744415225	2.1787721032
H	−3.1463895176	1.2108672517	1.5686491717
H	−6.3399637122	−2.8471907499	−1.425163324
H	−6.4101593702	−3.1315221401	0.3108028268
H	−8.7164624194	−1.9669554394	0.6929442638
H	−4.9829161348	1.7218669364	−3.2474931507
H	−4.6246870787	3.0313996466	−2.128038333
H	−7.577310548	1.8840045847	−3.0577647053
H	−4.8759687971	2.2617552658	2.9191155487
H	−5.3663618425	0.687102187	3.5335426529
H	−7.2795269463	3.0643330465	2.2685716527
H	0.2187397094	−3.3388730558	−0.3629195794
H	−2.1515076349	−2.803740537	−0.3623365916
H	−1.2156777642	1.3221360334	0.4225546094
H	1.1530667154	0.7730318854	0.4847036181
S	3.5524274855	−0.485825248	−0.2227215006
C	2.8903091381	−2.9041972859	0.3584422854
C	4.839710443	−1.6496405218	0.0310646248
H	2.3129977598	−3.7869501368	0.6108842654
C	4.3068162323	−2.8884036142	0.3249356927
C	6.238236156	−1.2930439622	−0.0888412239
H	4.9233501282	−3.7519123044	0.5491712631
C	7.2962044603	−2.1212641244	−0.4042552021
S	6.8169952573	0.3349806776	0.2116294984
H	7.1647181808	−3.1676062505	−0.6572979819
C	8.5505390618	−1.4618505838	−0.4134995725
C	8.4799766536	−0.117556028	−0.1159602604
H	9.479506199	−1.9563356016	−0.6758244805
C	9.5686306008	0.8623378365	−0.045253031
C	9.3441746021	2.2340092489	−0.2635877684
C	10.8806859943	0.442055181	0.2456467419
H	11.0723164489	−0.6072520829	0.4496124819
C	11.9275747391	1.3580842536	0.3027408334
H	8.3443816174	2.5820459352	−0.5091199776
C	10.3918772985	3.1495419323	−0.1943283889
H	10.1938762374	4.2037543048	−0.3686326104
C	11.6894972989	2.7172381009	0.0853546208
H	12.9316118355	1.0102686868	0.5309887862
H	12.5062322996	3.4318005833	0.1368210104
